# Practice patterns in transitioning patients from chronic kidney disease to dialysis: a survey of United States nephrologists

**DOI:** 10.1186/s12882-018-0943-0

**Published:** 2018-06-22

**Authors:** Mary C. Mallappallil, Steven Fishbane, Rimda Wanchoo, Edgar Lerma, Andrea Roche-Recinos, Moro Salifu

**Affiliations:** 1 Division of Nephrology, State University of New York at Downstate, 450 Clarkson Avenue, Box 52, Brooklyn, New York, 11203 USA; 2 Division of Nephrology, Hofstra Northwell School of Medicine, Manhasset, USA; 3 Division of Nephrology, University of Illinois at Chicago, Chicago, USA

**Keywords:** Blood pressure, Chronic kidney disease, Diuretic, Hemodialysis, Medication reconciliation, Nephrologist

## Abstract

**Background:**

There are no guidelines for transitioning patients from chronic kidney disease stage 5 to hemodialysis. We conducted this study to determine if there are uniform patterns in how nephrologists transition patients to dialysis.

**Methods:**

We designed an electronic survey with 39 questions and sent it to a database of practicing nephrologists at the National Kidney Foundation. Factors that were important for transitioning a patient to hemodialysis were evaluated, including medication changes on dialysis initiation, dry weight and dialysis prescription.

**Results:**

160 US Nephrologists replied to the survey; 18% (29/160) of the responses were completed via social media sites. Prior to dialysis, 74% (118/160), prescribed furosemide and 67% (107/160) used furosemide with metolazone. Once dialysis started, only 46% (74/160) of the responders continued patients on diuretics daily. Hypertension medications prescribed in dialysis were calcium channel blockers 69% (112/160), beta blockers 36% (58/160), angiotensin converting enzyme inhibitor 32% (53/160), angiotensin receptor blocker 29% (46/160) and diuretics 25% (42/160). Once dialysis started, 68% (109/160) routinely changed medications. Most, 67% (107/160) ordered patients to avoid anti-hypertensive medications on dialysis days to allow for ultrafiltration. Dry weight was determined in the first week by 29% (46/160) and in the first month by 53% (85/160). Most, 59% (94/160) felt that multiple causes lead to hypertension. Most nephrologists would prescribe small dialyzers and a shorter period of time for the first dialysis session.

**Conclusion:**

The transition period to chronic hemodialysis has variations in practice patterns and may benefit from further studies to optimize clinical practice.

**Electronic supplementary material:**

The online version of this article (10.1186/s12882-018-0943-0) contains supplementary material, which is available to authorized users.

## Background

According to the United States Renal Data System (USRDS), at the end of 2013 there were 661,648 prevalent patients with End Stage Renal Disease (ESRD); the unadjusted prevalence was 2034 per million in the U.S. population [1]. The first year after the initiation of hemodialysis (HD) is associated with increased mortality. The first 90 days has the highest risk which may be related to multiple causes [2]. Patterns from the Dialysis Outcomes and Practice Study reported the causes for mortality as cardiovascular disease (CVD), followed by withdrawal from dialysis; other factors include older age, catheter vascular access issues, albumin < 3.5 g/dL, phosphorus < 3.5 mg/dL, cancer and congestive heart failure (CHF) [3]. There is limited information about the practices related to the initiation of HD, which requires many clinical decisions and most of the literature is focused on the timing of initiation of HD.

Major contributors to CVD morbidity include hypertension and volume overload. There is little information about the ideal blood pressure (BP) in HD patients due to exclusion of this population in clinical trials; for example the trial of intensive versus standard blood pressure control, with a total of 9361 participants, of which 2646 had early chronic kidney disease (CKD) had no subjects who were on HD at the start of the study [4]. At present, the ideal blood pressure in a HD patients remains unclear and without defined BP targets [5–7] [https://www.kidney.org/sites/default/files/KDOQI-Clinical-Practice-Guideline-Hemodialysis-Update_Public-Review-Draft-FINAL_20150204.pdf] [8].

Multiple therapies including the attaining the correct dry weight, fluid and sodium restriction and medications to achieve BP control have been suggested [9–12]. Benefits of loop diuretics in HD patients include lower inter-dialytic weight gain, a lower odds of hyperkalemia, decrease in all cause and CVD mortality [13]. The benefits of renin angiotensin (RAS) blockade for CVD risk reduction is known, however, the data among HD patients is less certain [14]. A risk of combining both ACEI and ARB in HD that has been reported is an increase in both thirst and inter-dialytic fluid gain, while the variable risk of hyperkalemia was noted when RAS blockage was used [15–17]. Given the diverse findings, it is not surprising that a recent study noted that anti-hypertensive (anti-HTN) medications may be under-utilized in patients- once HD is initiated [18]. As there is no universally accepted technique to determine dry weight, the imprecise technique of altering the ultrafiltration goal aided by BP is used to attain volume control [19]. In the transition period it is unclear when the first dry weight should be determined.

The literature about the initial HD prescription with a shorter duration, lower blood flow and a smaller dialyzer to avoid disequilibrium is limited and comes from animal studies and case reports due to the difficulty of conducting such a trial in humans [20–22].

There is no uniform approach to adjust BP medications, diuretics or dry weight when a patient is started on HD. There is also no clear consensus regarding details on the initiation, the length of time of the first treatment blood flow and dialyzer to be used in HD. This study is designed to understand practice patterns in nephrologists with the goal of filling in the knowledge gaps in this area.

## Methods

We designed an electronic survey with 39 questions and sent it to a United States database of practicing nephrologists at the National Kidney Foundation (NKF). The survey was developed after a comprehensive literature search of the PubMed database on decisions about medication adjustments in early HD, initial HD prescription, volume and BP control in the HD treatment, failed to show clear evidence on clinical practice patterns. A data abstraction instrument with 39 questions in 7 domains including demographic, BP medication adjustment including ACE-I and ARB use, diuretic use, first HD prescription, access use, other modality use and presence of an ESRD educator was created. The questionnaire was validated prior to being sent out in a pilot test to a panel of 15 practicing nephrologist from multiple sites, including national experts in the field of transitioning patients from CKD to HD. The survey was modified based on the response process from this panel to ensure clarity of the questions.

The anonymous questionnaire is shown in Additional file 1 which is in the supplementary material. Institutional Review Board (IRB) approval was obtained from SUNY Downstate at Brooklyn. The survey was sent out via secured hyperlink (www.surveymonkey.com) twice by the NKF to its membership. An information sheet which explained the survey rationale and author contact information was attached to the questionnaire. Request to do the survey were posted on *Facebook* and *Twitter.* The survey was a voluntary without any remuneration for participation. The collected data was kept anonymous and aggregate data was analyzed using descriptive statistics, avoiding duplicate responses. Responses were in the form of single best choice; comparative situations and ranking. The sample frame was practicing US nephrologists and the response rate (< 5%) was in the range of other nephrology surveys [23]. No data was collected from the non-responders. Responses were collected between March 2015 and March 2016.

### Statistical analysis

The survey was conducted using *Surveymonkey.* All responses were via its secure link and all responses were available on its site as both individual anonymous and aggregate data. Electronic mail manager ensured no duplication of the survey from individuals. The survey had binary questions (more than/less than; yes/no) in addition to ranking scales (preferred choice with ranking 1 being the highest). The electronic questionnaire had multiple scroll-down pages that were easy to read with clear font. The survey had some questions where multiple options could be selected resulting in cumulative response rates exceeding 100%. Other questions had options to select from alternative mutually exclusive choices. Ranking was used to determine what was degrees of importance including “yes, likely, unlikely, no” and on a numeral scale to determine position of preferences. These questions enabled the computer algorithm to assign a unique weight for each choice. When data was summarized with descriptive statistics, it was described as weighted averages and as percentages.

## Results

Participants and their settings: Surveys were sent to members in the US-NKF nephrologist’s database in March 2015 and repeated again. The response rate was 160/ 5000 and responses were collected until March 2016. Eighteen percent (28/160) of the surveys that were completed, were in response to request placed on social media (*Facebook* and *Twitter*). Among the 160 responses, most 60% (96/160) were in practice for more than 10 years. Social media platforms were used by 28 nephrologist of whom 75% (19/28) were in practice for more than 10 years.

Among responders, 50% (80/160) started patients on HD in both inpatient or outpatient settings, where as 29% (46/160) reported starting patients on dialysis in the out-patient setting alone and 21% (34/160) reported that first dialysis in the hospital setting alone.

### Diuretic use

Asked about diuretic use the majority of the respondents 74% (118/160) reported using furosemide in CKD5 and 67% (107/160) reported using a combination of furosemide with metolazone. Multiple selections were allowed and choices were not mutually exclusive. Once HD had been started, 46% (74/160) of respondents continued to prescribe diuretics daily; 31% (50/160) ordered their use on non-HD days only and 22% (35/160) stopped the use of diuretics altogether. When asked about the use of spironolactone, 48% (77/160) of respondents reported that it was continued even after the initiation of HD (Table 1).

**Table 1:** Use of diuretics when transitioning a patient to hemodialysis

Diuretic use in CKD5	Total *N* = 160
Furosemide	118
Furosemide with metolazone	107
Diuretic use in CKD5-D	
Daily use once HD started	74
Non-HD day use	50
Stop diuretic when HD started	35

*CKD 5* = chronic kidney disease stage 5, *HD* = hemodialysis

### Anti-hypertensive effectiveness

Participants were asked about the most effective medications to control BP control in HD and the timing of the medications. The majority 69% (110/160) of the respondents considered calcium channel blockers to be the most effective; 34% (54/160) of them prescribed nifedipine and 35% (56/160) of them administered amlodipine. Beta blockers were reported to be effective by 36% (58/160); ACEI by 32% (51/160); ARB by 29% (46/160) and diuretics by 25% (40/160) (Table 2).

**Table 2:** Use of BP medications when transitioning a patient to hemodialysis

Blood pressure (BP) medication	*N* = 160
Calcium Channel blocker	110
Amlodipine	56
Nifedipine	54
Beta blockers	58
Angiotensin Converting Enzyme Inhibitor	51
Angiotensin Receptor Blocker	46
Diuretics	40
Avoid BP medications prior to hemodialysis to allow for ultrafiltration	107

*BP* = blood pressure

When asked what percentage of their HD patients are using ACEI/ARBs, 51% (82/160) of the respondents reported between30 to 60% (Table 3). When asked what factors were important in controlling BP in a patient on HD, 49% (78/160) responded that it was volume, 18% (29/160) replied that pre-existing HTN was important and 9% (13/160) replied that cardiac function was most important; the majority 59% (94/160) agreed it was a combination of factors that allow BP control.

**Table 3:** Use of Angiotensin Converting Enzyme Inhibitors and Angiotensin Receptor Blockers in Hemodialysis

Responses about ACEI and ARB on hemodialysis	*N* = 160
Report that between 30 and 60% of patients on ACEI or ARB	82
CKD5 would not stop ACE I or ARB	98
Would not change ACEI to one that is not dialyzed out	123
Would not stop ACEI or ARB despite hyperkalemia	104

### Timing of medication

Most of the respondents 68% (109/160) changed the dose of BP medications after the initiation of HD. In addition, 67% (107/160) of the respondents reported asking patients not to take their BP medications on the morning of HD to allow for greater ultrafiltration. When asked about details regarding the dose adjustments of the BP medications on a daily basis, 41% (66/160) of the respondents were more likely to tell the patients to take the BP medications daily and 41% (66/160) were more likely to tell the patient to take them on non-dialysis days only. Most of the respondents 58% (93/160) would not tell their newly started HD patients to stop the BP medications completely.

Regarding discontinuation of anti-hypertensive medications, in choosing between beta blockers and calcium channel blockers, 72% (115/160) would first stop calcium channel blockers. As far as medications being dialyzed out, 68% (109/160) or respondents would not change beta blockers to those were not dialyzed. After starting dialysis, most responders 69% (110/160) reported they had patients on once a day or long acting calcium channel blockers.

### First month of dialysis and changes

Ninety two percent of survey participants (147/160) replied that they would use a low blood flow setting (less than 300 ml/minute) for the first dialysis session and 48% (77/160) would use a smaller than usual dialyzer, while 94% (150/160) prescribed less time for the first session. In addition, 64% (102/160) would use a lower than usual dialysate flow rate. In general, 79% (126/160) of respondents would dialyze the patient for less than a full dialysis session at the first session (Table 4).

**Table 4:** Decision and changes made once hemodialysis was started

Decisions and changes made once HD started	*N* = 160
Less than usual time for first HD	150
Lower than usual blood flow for first dialysis	147
First anti-HTN medication to be stopped: calcium channel blocker	115
Changed BP medication or doses once HD started	109
Continued use of diuretics in HD daily	74
Continued use of daily anti-hypertensives	66
Medication review done every month	125
Selected choice that HTN in HD is caused by multiple factors	94

*HD* = hemodialysis, *ACEI* = Angiotensin Converting Enzyme Inhibitors, *HTN* = hypertension

In the first month of chronic dialysis, a large part of those surveyed, 88% (141/160), felt that ultrafiltration alone would help control BP and they would not use additional medications to the existing regiment. Fifty three percent (85/160) of responders would set the dry weight in the first month, 29% (46/160) in the first week and about 18% (29/160) would set the dry weight on the first day and adjust as needed.

Medication review was done one every month by the greater part of the participants 78% (125/160), every week by 12% (19/160) and as needed only by few 9% (15/160).

One option in non-adherent patients is to administer medications in dialysis. The larger part of those surveyed 85% (136/160) would not use BP medications while their patients were in HD even in the setting of non-adherence.

### Access use in early dialysis

In response to the time interval between access creation and maturation, 53% (85/160) of responders felt it was usually between 6 and 12 weeks, 34% (54/160) found it to be more than 12 weeks and 13% (21/160) noted that accesses would mature in less than 6 weeks.The question of how many angioplasties were considered to be acceptable before creating a new access had a distribution of responses: 27% (43/160) thought 3 angioplasties should be the most allowed, 25% (40/160) replied 2, 23% (37/160) replied more than 4, 18% (29/160) replied that only one angioplasty should be done before a new access was needed and 7% (11/160) thought 4 could be acceptable before primary access failure needing the creation of a new access. Forty two percent (67/160) of nephrologists responding to the survey felt that 30–60% of patients started HD with a permanent access in place, 31% (50/160) felt it was between 10 and 30% that started HD with a permanent access and 21% (34/160) replied that it was more than 60% of HD patients that started HD with the required access. Nine responders avoided this question.

### Other renal replacement therapy options

In the process of transitioning patients and other options being considered 44% (70/160) of responders felt that more than 50% of their incident HD patients who were eligible for transplant were listed for kidney transplantation. Eight percent of nephrologist who replied to the survey noted than < 10% of their CKD patients got a pre-emptive kidney transplantation. Asked about peritoneal dialysis (PD) as the first choice in those who were eligible, 44% (70/160) of respondents felt that only 10–50% started with their first choice even eligible to do so. The majority 65% (104/160) replied they had access to a patient educator.

## Discussion

The first 90 days after starting HD is a period of high mortality with CVD as the leading cause of death. Hypertension, left ventricular hypertrophy, electrolyte and electrocardiographic abnormalities in dialysis are known to be contribute to the high mortality in this population [24–28]. The transition period has changes in these risk factors notably: BP, electrolytes, ultrafiltration and medications. The gap in knowledge about optimal BP control in HD in the new HD patient makes clinical care complex during this period. As there is no unified approach for BP control in new HD patients this survey of practicing nephrologists to determine common practices is relevant.

The survey found the most effective medication in HD for BP control to be calcium channel blockers, followed by beta blockers, based on studies showing benefits of these agents in selected dialysis populations [29–32]. The effectiveness of calcium channel blocker in HD may be partly explained by the observation that the high inflow of calcium from the dialysate into the blood stream during HD may increase BP and would suggest that blocking the vascular smooth muscle would help better control BP [33].

The increasing use of ACE I and beta blockers in dialysis patients with comorbid conditions like congestive heart failure and the benefits thereof were noted in the literature and was reflected in the common use of these medications in this study [34]. For example studies of USRDS data noted that beta blockers use had increased 75% in those with previous acute myocardial infarctions and ACEI and ARB use had increased in those with CHF [35, 36]. In the survey, once on HD, the majority did not change ACEI (like enalapril, one third of which is dialyzed out) to one that would not be removed by dialysis, despite existing guidelines to do so. Most patients were instructed to avoid BP medications on HD days to allow for ultrafiltration- a common practice in HD, which is the opposite of recommendations that suggest daily nocturnal dosing of anti-hypertensive medications [37]. Avoidance of BP medications on HD days is not supported by literature; in one study of intra-dialytic BP variation it was noted that ultrafiltration volume resulted in greater change in systolic BP while the amount of anti-hypertensives was not associated with systolic BP changes [38]. A balanced option to minimize the risk of hypotension and cramping with HD while controlling BP is to keep patients on once a day nocturnal BP medications [39].

Responders noted that on HD initiation, anti-hypertensive medications were routinely changed, however uncertainty about BP fluctuations in the first month resulted in 88% using ultrafiltration alone to control BP without adding further medications. As dialysis vintage progressed there was a greater degree of comfort using anti-HTN medications.

The study found a majority of nephrologists prescribed diuretics in CKD 5 prior to HD but once HD was started less than half continued diuretics on a daily basis. In HD there is some benefit from diuretics; the underutilization noted in our study is consistent with known literature, possibly due to misperceived marginal benefits of the diuretics and possibly an attempt to lower the pill burden in new dialysis patients [40]. Further, with decreasing residual renal function, the effectiveness of diuretics decreases, while the risk of adverse drug events continues to be present. A practice of twice a week hemodialysis supplemented with diuretics may be of special benefit in the elderly with limited mobility to get to the HD unit. A recent study suggests that lower ultrafiltration in combination with a large dose diuretic use may help slow the decline in endogenous renal function for the first two years after dialysis initiation [41]. The majority of respondents felt that multiple causes lead to HTN in HD not just increased volume. Medication review was reported to be done monthly by the majority of nephrologists.

Volume appears to significantly contribute to HTN in HD and subsequently contribute to CVD mortality [19, 42, 43]. In the survey, nephrologists reported that dry weight was determined by between the first week and the first month. To combat inter-dialytic fluid gains, most ultrafiltration goals are determined clinically; only a minority of HD centers use bio-impedance routinely to determine fluid status according to a recent United Kingdom survey [44].

Most nephrologists would prescribe small dialyzers with a shorter period of time for the first dialysis session. The responses to questions about timing of access placement, maturity and salvage revealed the large majority considered access placement maturity needing at least 3 months, however the number of angioplasties to salvage the fistula showed a wide variation. Starting chronic dialysis is a major life sty le change for patients with many aspects that need improvement [45]. The presence of a dedicated dialysis educator may help make this transition smoother and 65% of the responders noted that their dialysis unit had an educator in their unit.

The limitations of our study includes the emphasis on the CVD risk in the transition period, while parameters like nutrition and phosphate control were not included [46]. The other limitation was the low response rate, but this similar to other nephrology studies in this group [47, 48]. As there is limited data about how nephrologists transition patients to dialysis, even with the above limitations, this study is helpful.

## Conclusion

In summary, transitioning patients from CKD5 to HD continues to remain challenging in terms of mortality, BP control, medication use, medication reconciliation and the time to determine initial dry weight of the patient. There is a lack of consistent patterns in how patients are transitioned from CKD5 to HD, which may be expected in the absence of strong data. Clinical trials are needed to help transitioning patients from CKD 5 to HD to help improve patient outcomes.

## Additional file

Additional file 1: Survey sample: transitioning patients from CKD to dialysis. (DOCX 16 kb)

## Abbreviations

ACEI: Angiotensin converting enzyme inhibitor
ARB: Angiotensin receptor blocker
BP: Blood pressure
CKD: Chronic kidney disease
CKD5 D: Chronic kidney disease on dialysis
CKD5: Chronic kidney disease stage 5
CVD: Cardio-vascular disease
eGFR: Estimated glomerular filtration rate
ESRD: End-stage renal disease
HD: Hemodialysis
NKF: National Kidney Foundation
USRDS: United States Renal Data System
