# Supplementary material for: Practice patterns in transitioning patients from chronic kidney disease to dialysis: a survey of United States nephrologists
Source: BMC Nephrol. 2018 Jun 22;19:147. doi: 10.1186/s12882-018-0943-0 (PMC6013940; doi:10.1186/s12882-018-0943-0)
Supplement: Supplementary file 1 — Survey sample: transitioning patients from CKD to dialysis. (DOCX 16 kb) [file 12882_2018_943_MOESM1_ESM.docx]

| **Appendix 1**  Practice Patterns in Transitioning patients from Chronic Kidney Disease to Dialysis: A Survey of United States Nephrologists |
| --- |

**1. How long have you been practicing Nephrology?**

a. Less than 10 years

b. More than 10 years

**2. Do you have dialysis patients in a for profit dialysis unit?**

a. yes

b. no

**3. Do you have patients in a hospital based dialysis unit?**

a. yes

b. no

**4. What is your preferred diuretic in CKD stage 5?**

a. furosemide

b. metolazone

c. spironolactone

d. hydrochlorothiazide

e. chlorthalidone

f. bumetinide

g.demedex

**5. What combination of diuretics in CKD 5 do you use the most?**

a. Furosemide with metolazone

b. Furosemide with hydrochlorothiazide

c. Furosemide with spironolactone

d. Metolazone with spironolactone

e. Bumetanide and metolazone

f. Demedex with metolazone

g. Other

**6. What is your rationale for using metolazone in CKD5?**

a. Just as potent as furosemide

b. More potent than furosemide

c. Longer half life

d. Less risk for Acute kidney injury

Other (please specify)

**7. When you start patients on dialysis how do you change the diuretics?**

a. Stop all together.

b. Continue it on non-dialysis days.

c. Continue to use it every day.

**8. Do you use spironolactone in patient on dialysis?**

a. Yes

b. No because it may cause hyperkalemia

c. No because of other side effects like gynecomastia

**9. Which of the following medications do you consider most effective in blood pressure control**

**in CKD5?**

a. Nifedipine

b. Amlodipine

c. Non-selective beta blockers eg Labetalol

d. Selective beta blockers like Metoprolol or atenolol

e. Angiotensin Converting Enzyme Inhibitors (ACEI)

f. Angiotensin Receptor Blockers (ARB)

g. diuretics

h. other

**10. Once the patient starts dialysis do you routinely change the dose of anti-HTN medications?**

a. Yes

b. no

**11. Do you ask patients to stop anti-HTN medications before dialysis on their dialysis days to**

**allow for greater ultrafiltration?**

a. yes

b. no

**12. In general, how do you change the dose of anti-HTN medications when a patient starts**

**dialysis?**

**Options: no // unlikely// likely // yes**

a. Tell patients to take the anti-htn medication daily-- no

a. Tell patients to take the anti-htn medication daily-- unlikely

a. Tell patients to take the anti-htn medication daily-- likely

a. Tell patients to take the anti-htn medication daily-- Yes

b. Tell the patient to take anti-htn medication on non-dialysis days only-- no

b. Tell the patient to take anti-htn medication on non-dialysis days only-- unlikely

b. Tell the patient to take anti-htn medication on non-dialysis days only-- likely

b. Tell the patient to take anti-htn medication on non-dialysis days only-- yes

c. Stop anti-htn medication altogether and restart them as needed-- no

c. Stop anti-htn medication altogether and restart them as needed -- unlikely

c. Stop anti-htn medication altogether and restart them as needed-- likely

c. Stop anti-htn medication altogether and restart them as needed—Yes

d. Stop medications onlyif SBP <140mmHg beforedialysis-- no

d. Stop medications only if SBP <140 mmHg before dialysis-- unlikely

d. Stop medications only if SBP <140 mmHg before dialysis-- likely

d. Stop medications only if SBP <140 mmHg before dialysis –yes

**13. On average, what percentage of your dialysis patients are on an ACE-I or ARB?**

a. less than 30%

b. 30-60%

c. more than 60%

**14. In general, once someone had CKD-5 do you reduce or stop the ACEI/ARB?**

a. Yes

b. No

**15. Do you routinely change ACEI in dialysis patients to those that cannot be dialyzed out once dialysis is initiated?**

a. yes

b. no

**16. In general, do you stop ACEI or ARB to control serum potassium in a dialysis patient?**

a. yes

b. no

**17. If you have to stop anti-hypertensive medications once the patient has started dialysis, which do you stop first- the calcium channel blockers or the beta blockers?**

a. Beta blockers

b. Calcium channel blockers

**18. Do you routinely switch to beta blockers that are not dialyzed once dialysis is initiated?**

a. Yes

b. No

**19. Do you routinely switch to once a day or long acting calcium channel blockers?**

a. Yes

b. No

**20. Once dialysis starts how frequently do you do a medication review with the patient?**

a. Every week

b. Every month

c. Once a year

d. As needed only

**21. Do you order patients to take their antihypertensive medications when they are in the dialysis unit to ensure adherence?**

a. Yes

b. No

**22. When starting chronic dialysis, usually, do you have the first session scheduled in the hospital or out- patient facility?**

a. In patient

b. Out patient

c. both

**23. Do you dialyze the patient for the first time with low blood flows <300 millilters/minute ?**

a. Yes

b. No

**24. Do you dialyze the patient for the first time with a smaller dialyzer than you usually use?**

a. Yes

b. No

**25. Do you prescribe less time than usual for the first hemodialysis session?**

a. yes

b. no

**26. For the first dialysis do you use lower dialysate flow rates than usual?**

a. yes

b. no

**27. Do you dialyze the patient for less than what you think would be the full dialysis time to prevent disequilibrium even if the BUN is less than 100mg/dL?**

a. yes

b. no

**28. In the first month of chronic dialysis, do you think patients need their BP controlled before they get put on the hemodialysis machine?**

a. Yes- if so do you use clonidine prior to HD to control BP

b. No- ultrafiltration will bring control BP

**29. When do you first establish a dry weight?**

a. First day and adjust as needed

b. About the first week

c. About the first month

**30. What factors do you consider to be the most important in controlling blood pressure?**

a. Volume

b. Cardiac function

c. Pre-existing hypertension

d. All of the above

**31. What percentage of your Hemodialysis patients get vein mapping before access creation?**

a. Less than 30%

b. Between 30-60%

c. more than 60%

**32. On average, what is the time interval between vein mapping and access creation?**

a. One week

b. One week to one month

c. More than one month

**33. On average what is the time interval between access creation and access maturation?**

a. 6 weeks

b. 6-12 weeks

c. >12 weeks

**34. After fistula creation how many angioplasties do you generally find to be acceptable prior to**

**creating a new access.**

1. One

2. Two

3. Three

4. Four

5. More than 4

**35. What percentage of your patients have a permanent access that is ready when they start**

**dialysis?**

a. <10%

b. 10-30%

c. 30-60%

d. >60%

**36. What percentage of your patients who are eligible are listed for transplant?**

a. less than 10%

b. between 10-50%

c. more than 50%

**37. What proportion of your CKD patients get a preemtive transplant?**

a. <10%

b. 10-30%

c. >30%

**38. On average what percentage of your patients who meet criteria for peritoneal dialysis get PD as their first modality for dialysis?**

a. less than 10%

b. 10-50%

c. between 50% to 90%

d. >90

**39. Do you have a dedicated patient educator/ ESRD co-ordinator?**

a. yes

b. no
